# Molecular characterization of a *Trichinella spiralis* aspartic protease and its facilitation role in larval invasion of host intestinal epithelial cells

**DOI:** 10.1371/journal.pntd.0008269

**Published:** 2020-04-27

**Authors:** Jia Xu, Ruo Dan Liu, Sheng Jie Bai, Hui Nan Hao, Wen Wen Yue, Yang Xiu Yue Xu, Shao Rong Long, Jing Cui, Zhong Quan Wang

**Affiliations:** Department of Parasitology, Medical College, Zhengzhou University, Zhengzhou, PR China; Istituto Superiore di Sanità, ITALY

## Abstract

**Background:**

*T*. *spiralis* aspartic protease has been identified in excretion/secretion (ES) proteins, but its roles in larval invasion are unclear. The aim of this study was to characterize *T*. *spiralis* aspartic protease-2 (TsASP2) and assess its roles in *T*. *spiralis* invasion into intestinal epithelial cells (IECs) using RNAi.

**Methodology/Principal findings:**

Recombinant TsASP2 (rTsASP2) was expressed and purified. The native TsASP2 of 43 kDa was recognized by anti-rTsASP2 serum in all worm stages except newborn larvae (NBL), and qPCR indicated that TsASP2 transcription was highest at the stage of intestinal infective larvae (IIL). IFA results confirmed that TsASP2 was located in the hindgut, midgut and muscle cells of muscle larvae (ML) and IIL and intrauterine embryos of the female adult worm (AW), but not in NBL. rTsASP2 cleaved several host proteins (human hemoglobin (Hb), mouse Hb, collagen and IgM). The proteolytic activity of rTsASP2 was host-specific, as it hydrolyzed mouse Hb more efficiently than human Hb. The enzymatic activity of rTsASP2 was significantly inhibited by pepstatin A. The expression levels of TsASP2 mRNA and protein were significantly suppressed by RNAi with 5 μM TsASP2-specific siRNA. Native aspartic protease activity in ML crude proteins was reduced to 54.82% after transfection with siRNA. Larval invasion of IECs was promoted by rTsASP2 and inhibited by anti-rTsASP2 serum and siRNA. Furthermore, cell monolayer damage due to larval invasion was obviously alleviated when siRNA-treated larvae were used. The adult worm burden, length of adult worms and female fecundity were clearly reduced in mice challenged using siRNA-treated ML relative to the PBS group,

**Conclusions:**

rTsASP2 possesses the enzymatic activity of native aspartic protease and facilitates *T*. *spiralis* invasion of host IECs.

## Author summary

Trichinellosis has been regarded as a re-emerging or emerging disease, and it is distributed worldwide. Studies investigating *T*. *spiralis* ES protein are beneficial to explore potential molecular targets for anti-*T*. *spiralis* vaccines. The functions of aspartic protease have been studied in other parasites and demonstrated to be crucial for their parasitism in the host. However, the functions of *T*. *spiralis* aspartic protease have not been reported. Here, we expressed and purified a *T*. *spiralis* aspartic protease-2 (TsASP2). Our results showed that TsASP2 was expressed in all *T*. *spiralis* developmental stages other than NBL and located in the hindgut, midgut and muscle cells of ML and IIL, as well as in areas surrounding embryos within the female uterus. The rTsASP2 possessed aspartic protease activity and functioned to cleave hemoglobin, collagen and IgM. Silencing of the *TsASP2* gene could significantly decrease the aspartic protease activity in muscle larva crude proteins, larval invasion of IECs and worm development in the host. We conclude that TsASP2 plays an important role in *T*. *spiralis* penetration into host intestinal epithelial cells and could be a candidate vaccine target molecule against *T*. *spiralis* infection.

## Introduction

As a pathogen of worldwide food-borne zoonosis, *Trichinella* parasite has been found to infect more than 100 mammalian species [1]. Humans acquire trichinellosis through the ingestion of raw or poorly cooked meat containing the infective larvae of *Trichinella* [2]. Outbreaks of trichinellosis have been reported in many counties worldwide, especially in developing countries [3,4]. In China, 15 outbreaks were recorded from 2004 to 2009 due to raw or undercooked pork food [5]. Trichinellosis has not only become a public health concern but also threatened porcine animal production and food safety [6]. Thus, trichinellosis has been regarded as a re-emerging or emerging disease worldwide and has gained increasing attention [7]. These concerns have promoted the exploration of anti-*Trichinella* vaccines, especially to identify molecules that play key roles in *T*. *spiralis* invasion of intestinal epithelium [8].

Muscle larvae (ML) of *T*. *spiralis* dwell in skeletal muscles of hosts. When the contaminated meat is ingested, the ML are liberated from the muscles by digestive enzymes in the stomach [9]. After activation by bile and enteral contents, the larvae develop into intestine infective larvae (IIL). The IIL invade the small intestinal epithelium where they undergo four molts to mature into adult worms (AW). The newborn larvae (NBL) are shed by female AW after mating and then enter the venules and lymphatic vessels, eventually penetrating into the skeletal muscle via the bloodstream [10]. The mechanism of *T*. *spiralis* penetration into intestinal epithelium is critical for *T*. *spiralis* to complete its lifecycle in the host and seems to be orchestrated by several *T*. *spiralis* protein molecules. Thus, studies on the characterization and functions of these *T*. *spiralis* proteins will be very valuable for the development of an anti-*Trichinella* vaccine.

Proteinases released by *T*. *spiralis* play an essential role in parasite invasion [11,12]. Several serine proteinases have been identified and demonstrated to be involved in various adaptive functions, such as tissue invasion and immune evasion, [13–15]. In another study, proteinases produced by *T*. *spiralis* adult worms could cleave fibrinogen and plasminogen, and this hydrolytic activity might be related to the activity of a serine or aspartyl proteinase [16]. These studies revealed that the characterization of proteinases derived from *T*. *spiralis* would provide critical information for explorations of anti-*T*. *spiralis* vaccines.

Aspartic protease, classified as clan AA in the MEROPS database, was the first protease type to be described [17]. The aspartic protease family, including pepsins, renins, cathepsins D and E, and chymosins [18], is characterized by a typical Asp-Thr (Ser)-Gly sequence, and the protein hydrolytic activity is closely associated with an Asp residue in the clefts of the active sites [19]. The proteolytic function of aspartic proteases is optimal under acidic conditions (pH 3.0–4.0) [12]. Many aspartic proteases have been demonstrated to play key roles in the degradation of host hemoglobin and other proteins, especially in hematophagous parasites. An aspartic protease named Na-APR-1/2 originating from hookworms can efficiently cleave hemoglobin, collagen and serum albumin from human and dog [20,21]. A cathepsin D-like aspartic protease from *Opisthorchis viverrini* has been shown to digest bovine serum albumin (BSA) and hemoglobin [22]. The proteolytic activity and probable functions of other parasites such as *Schistosoma mansoni*, *Necator americanus* and *Onchocerca volvulus*, which can also secrete aspartic protease, have also been investigated [23–25]. An aspartic protease has been identified in *T*. *spiralis* ES proteins [11], but its function in *T*. *spiralis* invasion into intestinal epithelium is not clear.

Since it was first conducted in *Caenorhabditis elegans* [26], RNA interference (RNAi) has been widely applied to identify gene function in parasites. The application of RNAi to downregulate target molecules to reduce protein expression can affect specific gene functions during some developmental stages of parasites. Recently, RNAi was used to identify some important protein functions of parasites, such as *Clonorchis sinensis* enolase [27], ATPase RNA helicase and trehalose-6-phosphate phosphatase in *Brugia malayi* [28,29], nematode *Setaria digitata*-specific protein [30], and calcium-regulated heat-stable protein of 24 kDa and type V collagen in *Schistosoma japonicum* [31,32]. Nevertheless, the functions of only a few *T*. *spiralis* genes have been ascertained by RNAi, including the Nudix hydrolase, serine protease inhibitor (TsSPI) and paramyosin genes [7,33,34].

Four aspartic proteases were identified in the draft genome of *T*. *spiralis*, all of which are bilobal enzymes. The similarities among four aspartic proteases ranged from 15.6% to 81.8%. However, only *T*. *spiralis* aspartic protease 2 (TsASP2; GenBank: 339237490) from a *T*. *spiralis* muscle larva cDNA library has been demonstrated to be present in *T*. *spiralis* excretion/secretion (ES) proteins [11]; however, its function has remained unclear. Since the proteases in ES proteins are first exposed to host intestinal epithelium cells (IECs), they are likely to play a major role or participate in larval invasion of IECs. Therefore, TsASP2 was selected and expressed in the present study. We further investigated the functions of TsASP2 in *T*. *spiralis* larval invasion of host IECs, and a specific TsASP2 siRNA sequence was designed and electroporated into muscle larvae to elucidate the gene functions.

## Methods and materials

### Ethics statement

This study was carried out according to the National Guidelines for Experimental Animal Welfare (Minister of Science and Technology, People’s Republic of China, 2006). The animal experiment procedure was approved by the institutional Life Science Ethics Committee, Zhengzhou University (No. SCXK 2017–0001).

### Parasites, experimental cells and animals

The *T*. *spiralis* isolate (ISS534) used in this study was originally obtained from domestic pigs in Nanyang (Henan province, China) and maintained in our laboratory by serial passages in BALB/c mice. Specific pathogen-free (SPF) BALB/c mice aged 5 weeks old were purchased from the Experimental Animal Center of Henan Province. Normal IECs were obtained from mouse small intestines and used for the invasion assay at passage 8 [10]. The IECs were cultured as previously described [35].

### Worm collection and protein preparation

*T*. *spiralis* ML were collected from infected mice at 42 days post-infection (dpi) by digestion of carcasses with 0.75% pepsin and 1% HCl as previously described [36]. The IIL were obtained from small intestines of infected mice at 6 hours post-infection (hpi). AW were isolated from duodenum and jejunum of infected mice at 3 and 6 dpi. The newborn larvae (NBL) were obtained from female adult worms cultured as previously described [37]. The ML ES antigens and crude soluble antigens of AW, NBL, ML and IIL were prepared as previously reported [10]. Briefly, the worms were first homogenized using a high-speed tissue grinder (KZ-II Servicebio) for 1 min, and the worm fragments were further homogenized by ultrasonication (99 3-s cycles, 100 W, 0°C). The supernatant containing crude proteins was collected after centrifugation at 15,000 *g* for 1 h at 4°C. To collect the ES proteins, the larvae were washed with sterile saline and then cultured in RPMI-1640 medium at a density of 5000 worms/ml for 18 h at 37°C in 5% CO_2_. After the media containing ES proteins were filtered with a 0.22-μm membrane, the ultrafiltration tubes were used to concentrate the ES proteins. The concentration of these proteins was measured using the Bradford method.

### Expression of recombinant TsASP2 protein in *Escherichia coli*

The entire CDS sequence of TsASP2 encoding aspartic protease (spanning Gly-17 to Ser-406) without the signal peptide was amplified by PCR and cloned into the expression vector pQE80L (His tag) and pMAL-c2x (MBP tag) using the *Bam* HI and *Hin*d III site. The recombinant plasmids were transferred into BL21 (DE3). The rTsASP2 was induced with 0.1 mM IPTG at 16°C for 20 h. The Ni-NTA-Sefinose resin (for His-tagged protein) and Amylose Pre-packed Column (for MBP-tagged protein) (NEB, China) were used to purify the rTsASP2. The rTsASP2 with a His tag was purified under denaturing conditions and then refolded and used in subsequent immunization experiments. The rTsASP2 with an MBP tag was used for its functional characterization. SDS-PAGE was applied to analyze the purified rTsASP2, and the Bradford method was used to determine the rTsASP2 concentration.

### Preparation of anti-rTsASP2 serum

Fifteen BALB/c mice were used to produce anti-rTsASP2 serum. First, the mice were immunized subcutaneously with 20 μg rTsASP2 emulsified with complete Freund’s adjuvant. Three boost immunizations were further carried out every 2 weeks by injecting the same amount rTsASP2 emulsified with incomplete Freund’s adjuvant. Blood samples were collected from immunized mice on day 7 after last immunization, and sera were isolated.

### Western blot analysis

Western blot analysis was carried out according to previous studies [38]. First, ES protein and crude protein samples from ML, IIL, AW and NBL were separated by SDS-PAGE on a 12% acrylamide separation gel and subsequently transferred onto polyvinylidene difluoride (PVDF) membrane. Second, the membranes were blocked with 5% skim milk in Tris-buffered saline containing 0.05% Tween-20 (TBST). The membranes were washed three times with TBST to remove the residual skim milk and then incubated with 1:100 dilutions of anti-rTsASP2 serum at 37°C for 1 h. Following another wash with TBST, the membranes were incubated with 1:5 000 dilutions of HRP-conjugated goat anti-mouse IgG (Southern Biotechnology, USA) at 37°C for 1 h. Finally, the membranes were stained with 3, 3'-diaminobenzidine tetrahydrochloride (DAB; Sigma) as a substrate, which was terminated by washing the membranes with deionized water [35,39].

### qPCR

Total RNAs of different *T*. *spiralis* phases (ML, IIL, 3-day AW, 6-day AW and NBL) were extracted using TRIzol reagent (Invitrogen, Carlsbad, CA, USA). All RNA samples were pre-treated with DNase I (Thermo Fisher Scientific, San Francisco, CA, USA) before use. Transcription of the TsASP2 gene at different worm stages was measured by qPCR as previously described [40]. The qPCR experiment was performed on a 7500 Fast Real-time PCR System (Applied Biosystems). The specific primers (across introns) for TsASP2 gene amplification included forward 5'-AATTCAACCCGTCCGTCTCC-3' and reverse 5'-TTCCAACTTGCGGCCATAGT-3'. The TsASP2 transcription level was normalized by subtracting the transcription level of GAPDH (GenBank: AF452239). The data were calculated according to the comparative Ct (2^-ΔΔCt^) method [40]. Each experiment was performed with three replicates of each sample.

### Immunofluorescent assay (IFA)

IFA was carried out to confirm the expression of TsASP2 at diverse *T*. *spiralis* stages. Various *T*. *spiralis* worm stages (ML, IIL, AW and NBL) were fixed in paraformaldehyde and embedded in paraffin. The 2-μm-thick sections were prepared using a microtome. After blocking with 5% normal goat serum, the sections were incubated with anti-rTsASP2 serum (1:50 dilutions) at 37°C for 1 h. After washing with PBS, they were incubated with FITC-labeled goat anti-mouse IgG (1:100 dilution, Santa Cruz, USA) and observed under a fluorescence microscope (Olympus, Japan) [41].

### Cleavage of Hb and other proteins by rTsASP2

Hemoglobin (Hb) from mice and human was collected by lysis of fresh erythrocytes as previously described [20]. Approximately 2 μg Hb was incubated with 0.8 μg rTsASP2 in pH 2.5–5.5 buffer solution, and the hydrolysates were detected by SDS-PAGE and staining with Coomassie brilliant blue. To compare the hydrolysis efficiency of rTsASP2 for different Hb, hydrolysis experiments were further conducted with different incubation times (5 min, 30 min, 90 min and 4 h). Other proteins (collagen IV, human IgM and IgG) were also used as substrates to evaluate the cleavage function and specificity of rTsASP2. Anti-rTsASP2 serum (heated at 56°C for 35 min or not heated) at a 1:25 dilution or 0.8 μg pepstatin A was pre-incubated with rTsASP2 for 1 h, followed by incubation with mouse Hb for 2 h to detect the enzyme activity.

### Enzymatic activity of rTsASP2

The fluorescent substrate MCA-Gly-Lys-Pro-Ile-Leu-Phe-Phe-Arg-Leu-Lys (DNP) -D-Arg-amide (synthesized by Sangon, Shanghai) was used to assess the enzymatic activity of rTsASP2 [42]. The total reaction volume was 100 μl, including 20 μg/ml rTsASP2 and 5 μM fluorescent substrate. After the enzyme and substrate were mixed for 30 min, the reaction termination fluid (35% methyl alcohol, 30% ethyl alcohol, 35% ddH_2_O) was added, and the fluorescence intensity was continuously detected by spectrophotofluorometry (Synergy H1, BioTek, USA) using an excitation wavelength of 320 nm and emission wavelength of 390 nm, respectively. To determine the optimal pH, the reaction was carried out using assay buffers with different pH values: 0.2 mol/L Gly-HCl buffer (pH 2.0–3.0), 0.2 mol/L HAc-NaAc (pH 3.5–5.5), 0.2 mol/L Na_2_HPO_4_-NaH_2_PO_4_ (pH 6.0). The relative enzymatic activity was calculated by setting the highest enzyme activity as 100% relative activity. Different concentrations (1 mM, 10 mM, 100 mM, and 200 mM) of Fe^2+^, Zn^2+^, Cu^2+^, Mn^2+^ and Mg^2+^ were added to the assay buffer to evaluate the influence of metal ions on rTsASP2 enzyme activity. Inhibitors (pepstatin A, PMSF, 1, 10-phenanthrolin, AEBSF, EDTA and E-64) were pre-incubated with rTsASP2 for 30 min, and then the effects on rTsASP2 enzyme activity were evaluated. Reaction buffers without addition of metal ions and inhibitors were used as respective controls.

### Electroporation of *T*. *spiralis* ML with siRNA

The TsASP2-specific siRNA was designed using siDirect version 2.0 according to the complete cDNA encoding TsASP2. Three siRNA sequences were used in the present study, including a TsASP2-specific siRNA, a control siRNA carrying the scrambled sequence and another *T*. *spiralis* aspartic protease-1 (TsASP1) siRNA sequence to control for specificity. All siRNAs were synthesized by Sangon Biotech (Shanghai, China), and the sequence information is listed in Table 1.

**Table 1:** The sequences of the siRNAs.

siRNA’ name	sense(5'-3')	antisense(5'-3')
**TsASP1- siRNA**	GUCAACAUUCAAAGAAUAUTT	AUAUUCUUUGAAUGUUGACTT
**TsASP2- siRNA**	CAUGAUUGAGCAAAAUCUUTT	AAGAUUUUGCUCAAUCAUGTT
**Control siRNA**	AUCGGCUACCAAGUCAUACTT	GUAUGACUUGGUAGCCGAUTT

The ML was obtained from infected mice at 35 dpi and washed three times with PBS. Approximately 2500 ML worms were treated with 5 μM siRNA in electroporation buffer. The siRNA was delivered into ML by electroporation (125 V, 20 ms) with a Gene Pulse Xcell System (Bio-Rad, USA), after which the worms were cultured in DMEM for 1–7 days.

### Analysis of TsASP2 mRNA and protein expression after siRNA transfection

qPCR was performed to analyze TsASP2 mRNA transcription in siRNA-treated ML as described above. The TsASP2 protein expression in treated worms was also evaluated by western blot analysis [34]. In brief, the crude proteins extracted from siRNA-treated ML were separated by SDS-PAGE and then transferred onto a PVDF membrane. Anti-rTsASP2 serum (1:100) was first used to recognize the membrane and then visualized using an enhanced chemiluminescent kit (Beyotime Biotech, China). The membrane was washed with stripping buffer (Beyotime Biotech, China) and then incubated with mouse anti-GAPDH IgG for quantitative protein control.

### RNAi effect on the enzymatic activity of aspartic protease

Crude protein extracts were obtained from approximately 2000 ML treated with siRNA or PBS. The enzymatic activity assay was carried out in a 100-μl reaction mixture with 100 μg crude protein and a final concentration of 5 μM fluorogenic substrate in sodium format, pH 3.5 and incubated at 37°C for 30 min. After the reaction was terminated, the fluorescence from substrate hydrolysis was measured as described above.

### RNAi effect on *in vitro* larval penetration

The effect of RNAi on *in vitro* IEC penetration by *T*. *spiralis* was also assessed. C2C12 was insensitive to *T*. *spiralis* penetration and used as negative control. The rTsASP2 protein was found to facilitate *in vitro T*. *spiralis* invasion of IEC. Briefly, the ML were activated into IIL with 5% swine bile, and the IEC cell monolayers (grown to confluence in 6-well culture plates) were overlaid with 100 IIL mixed with 2 ml of DMEM semisolid medium [10]. Different concentrations of rTsASP2 proteins were added to the medium to investigate the effects of the rTsASP2 protein on larval invasion. The different dilutions (1:50–1:800) of anti-rTsASP2 serum, infection serum and normal serum were then added to the medium. The IILs that had penetrated into the IECs were counted by microscopy after being cultivated for 2 h at 37°C. The penetrated and unpenetrated worms were examined and counted as previously reported [10,34]. Subsequently, the IEC cell monolayer capped by 100 RNAi treated or untreated larvae was also observed to evaluate the RNAi effect on larval invasion. The larval invasion rate was compared between experiments, and the larval invasive ability was assessed.

To confirm the RNAi effect on larval invasion, dead or damaged cells were also counted [43]. Briefly, after incubation, monolayers were stained with 10 μg/ml propidium iodide (PI) for 10 min and washed three times with PBS. The numbers of dead or damaged cells (stained red) were determined by fluorescence microscopy (Olympus, IX53, Japan) and NIH Image software. Dead or damaged cells was counted for 3 monolayers for each group. A total of 10 microscope fields of each monolayer were captured, and the mean number of dead (damage) cells was determined.

To detect the remaining TsASP2 in monolayers, the IECs were grown to confluence on coverslips. Following incubation, the slide was first stained with PI and then fixed with 4% paraformaldehyde for 15 min. The monolayers were probed using anti-rTsASP2 serum (1:20 dilutions) for 1 h at 37°C. Positive and negative controls were also probed using infection serum and normal serum instead of anti-rTsASP2 serum. After washing three times with PBS, the coverslips were incubated with FITC-conjugated goat anti-mouse IgG (1:100) for 1 h at 37°C. Next, the coverslips were rinsed three times with PBS and mounted for further fluorescence microscopy observation.

### RNAi effect on larval development and survival

To evaluate the infectivity of siRNA-treated larvae, the larval challenge infection experiment was performed. Thirty mice were equally divided into 3 groups, and each mouse in the different groups was orally infected with 300 larvae treated with TsASP2 siRNA, control siRNA or PBS. Adult worms at 6 dpi were collected from each group, and the parasite burden was ascertained. The fecundity of female AW was assessed by counting the newborn larva production by each female worm for 72 h. The morphology of AW and NBL was observed and imaged under a microscope (OLYMPUS IX53), and the length of the worms was measured using the measuring tool provided with the image software (CellSens Standard).

### Statistical analysis

Data analysis was performed with the aid of SPSS 19.0 software. The data are presented as the mean ± standard deviation (SD). The Chi square test was used to compare the percentage of larval invasion in the different groups. One-way ANOVA was used to compare the data from different groups in the following experiment: the relative TsASP2 transcription or expression levels, enzymatic activity of aspartic protease from siRNA-treated larvae, length of worms and damaged cell numbers of IEC destroyed by *T*. *spiralis*. *P* < 0.05 was regarded as a statistically significant difference.

## Results

### Expression of rTsASP2

**Fig 1:**
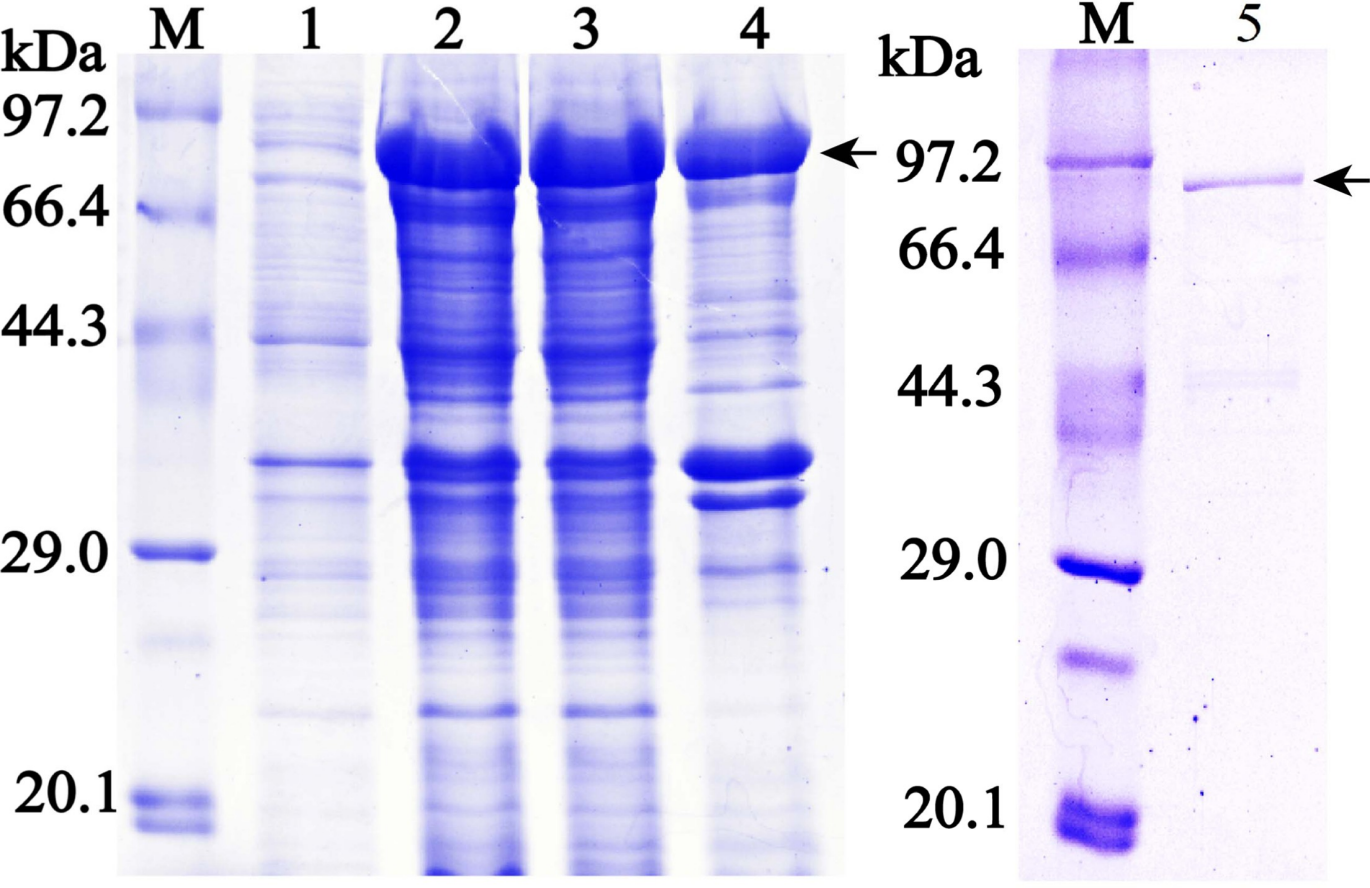
SDS-PAGE analysis of rTsASP2. M: protein marker; lane 1: lysates of recombinant bacteria incorporating pMAL-C2X/TsASP2 without induction; lane 2: lysates of recombinant bacteria incorporating pMAL-C2X/TsASP2 after induction; lane 3: lysate supernatant of recombinant bacteria incorporating pMAL-C2X/TsASP2 after induction; lane 4: sediment of recombinant bacteria incorporating pMAL-C2X/TsASP2 after induction; lane 5: purified rTsASP2. The arrow represents the band of rTsASP2 (86.4 kDa).

The 1170-bp CDS sequence without a signal peptide of TsASP2 was amplified, which encodes 406 amino acids. After BL21 (DE3) containing the two different recombinant plasmids (pQE-80L/TsASP2 and pMAL-c2x/TsASP2) was induced with IPTG, the fusion protein was expressed as 43.4 kDa (His tag) (S1 Fig) for the former recombinant protein and 86.4 kDa (containing the 43 kDa MBP tag) for the other one (**Fig 1**).

### Western blot and qRT-PCR analysis of TsASP2 expression in various stages

**Fig 2:**
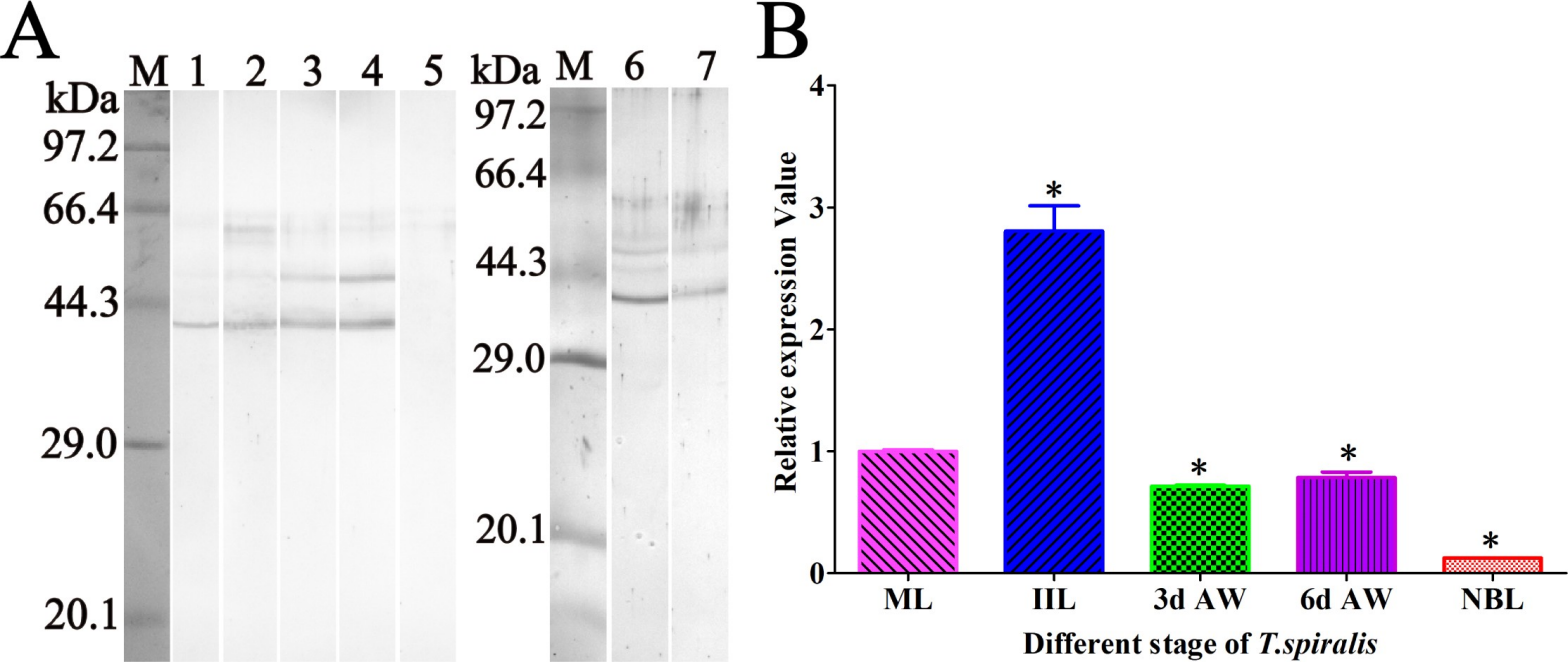
**Western blot (A) and qPCR (B) analysis of TsASP2 protein and mRNA expression in different *T*. *spiralis* stages. A:** Anti-rTsASP2 serum recognized native TsASP2 in different *T*. *spiralis* stage crude proteins, including ML (lane 1), 6-h IIL (lane 2), 3-d AW (lane 3), 6-d AW (lane 4), but not NBL (lane 5), and ML ES (lane 6) and IIL ES (lane 7). **B:** The TsASP2 mRNA expression levels in different *T*. *spiralis* stages were assessed by qPCR. Asterisks indicate a statistically significant difference compared with the ML stage (*P < 0.05).

Western blot analysis showed that the native TsASP2 protein of 43 kDa was recognized by anti-rTsASP2 serum **(Fig 2A)** in all worm stages except NBL, and the other bands that were also recognized may have been protein isoforms of TsASP2. TsASP2 gene transcription in these stages was further detected by qPCR. The results showed that the transcription level of TsASP2 gene was highest at the IIL stage and lowest at the NBL stage **(Fig 2B)**. The TsASP2 transcription level was significantly higher in the IIL stage than the other stages (*F* = 3.719, *P* < 0.01), while the transcription level was significant lower in NBL stage than the other stages (*F* = 4.007, *P* < 0.0001). The relatively low expression of the TsASP2 gene in the NBL stage could explain why TsASP2 protein in NBL proteins was not recognized by anti-TsASP2 serum by western blot analysis.

### Expression and location of TsASP2 in different stages

**Fig 3:**
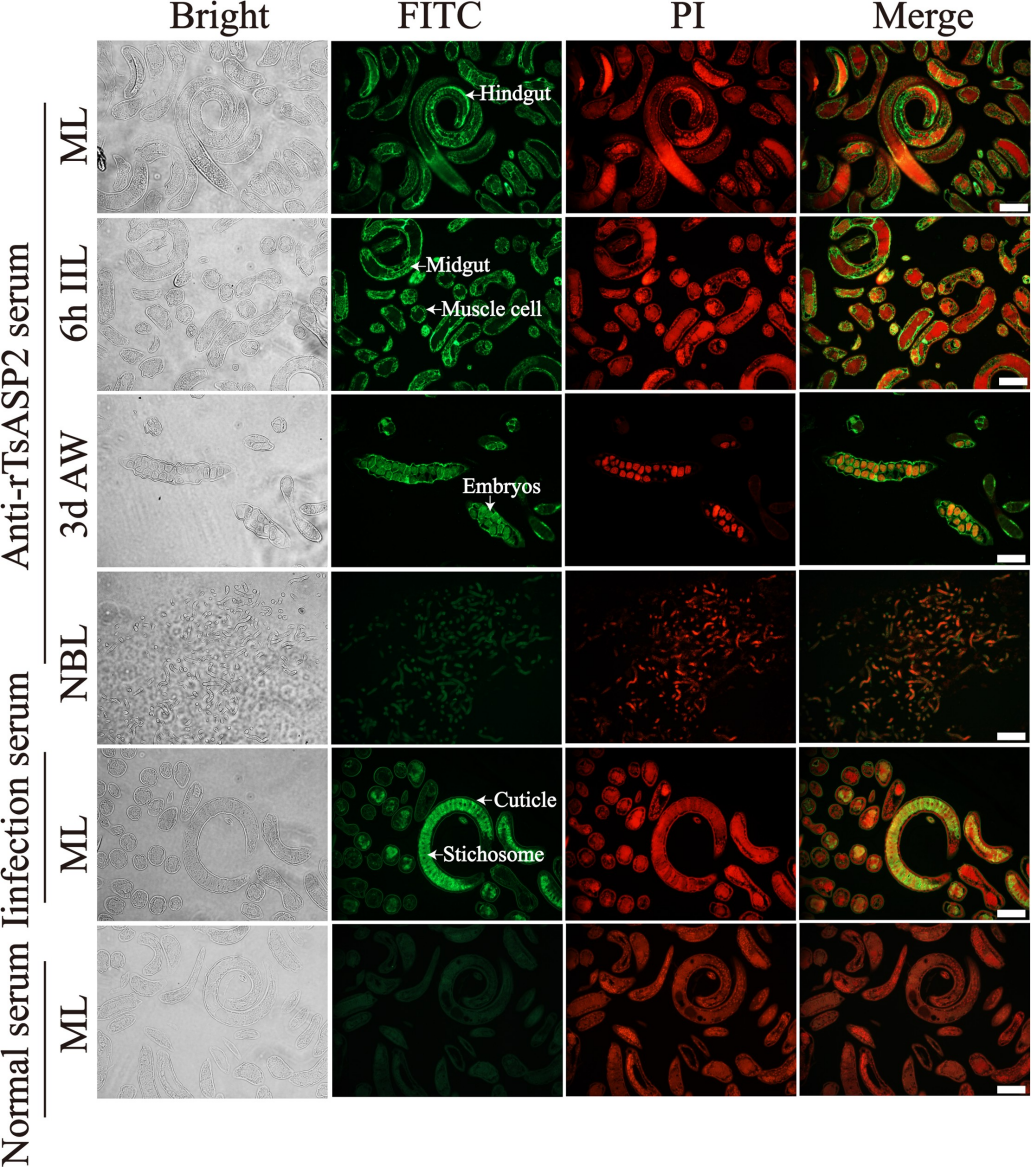
Immunofluorescent analysis of TsASP2 location in various worm phases. Intense green staining was observed in the midgut, hindgut and muscle cells of ML and 6-h IIL, as well as around intrauterine embryos of female adults; no immuno-staining was observed in NBL; ML recognition by infection serum as a positive control and normal serum as a negative control. The cell nuclei were stained red with propidium iodide (PI). Scale bars: 50 μm.

The IFA results confirmed the expression of TsASP2 in different life cycle stages of *T*. *spiralis*. Immunofluorescent staining of hindgut, midgut and muscle cells of ML and IIL was observed, as well as around the embryos of AW, but not NBL (**Fig 3**).

### Cleavage of different protein by rTsASP2

**Fig 4:**
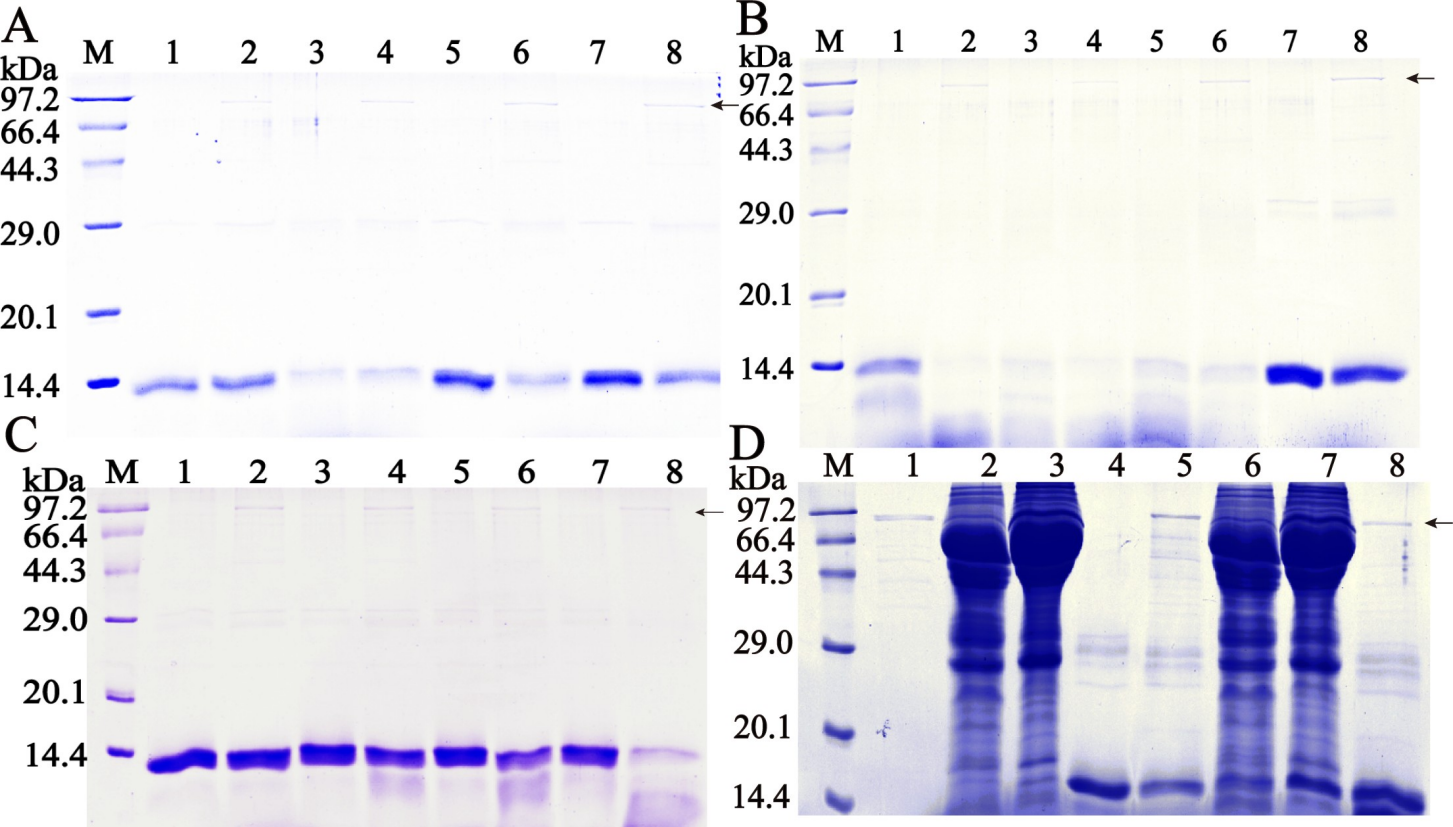
Hemoglobin degradation. **A-B** Hydrolysis of Hb by rTsASP2 at different pH values. **A**: human Hb; **B**: mouse Hb; M: protein marker; lanes 1, 3, 5 and 7: Hb alone; lanes 2, 4, 6 and 8: Hb+ rTsASP2; lanes 1 and 2: pH 2.5; lanes 3 and 4: pH 3.5; lanes 5 and 6: pH 4.5; lanes 7 and 8: pH 5.5. **C:** Hydrolysis efficiency effect of rTsASP2 on mouse Hb (pH 2.5). M: protein marker; lanes 1, 3, 5 and 7: Hb alone; lanes 2, 4, 6 and 8: Hb+ rTsASP2; lanes 1 and 2: 5 min; lanes 3 and 4: 30 min; lanes 5 and 6: 90 min; lanes 7 and 8: 4 h**. D:** Inhibition effect of anti-rTsASP2 serum and pepstatin A on rTsASP2 hydrolysis of mouse Hb (pH 2.5). M: protein marker; lane 1: rTsASP2; lane 2: anti-rTsASP2 serum; lane 3: heated anti-rTsASP2 serum; lane 4: Hb; lane 5: Hb +rTsASP2; lane 6: anti-rTsASP2 serum pre-incubated with rTsASP2 +Hb; lane 7: heated anti-rTsASP2 serum pre-incubated with rTsASP2+ Hb; lane 8: pepstatin A pre-incubated with rTsASP2 + Hb. The arrow represents the band of rTsASP2 (86.4 kDa).

As Hb protein has frequently been used as a substrate to investigate the proteolytic roles of aspartic protease, the enzymatic activity of rTsASP2 was first confirmed by the cleavage of Hb protein from human and mouse. The results showed that human and mouse Hb were hydrolyzed by rTsASP2 at pH 2.5–5.5. Hb could be degraded by self-hydrolysis at an acidic pH, especially for mouse Hb at pH 2.5–4.5. After comparison to the patterns of self-hydrolysis at the same pH, the optimal pH was determined for the degradation of different Hbs. rTsASP2 could degrade mouse Hb most efficiently at pH 2.5, while it degraded human Hb at an optimal pH of 4.5. Furthermore, the degradation efficiency of Hbs hydrolyzed by rTsASP2 was observed (**Fig 4**). Degradation of mouse Hb was detected at 30 min (lane 4) after incubation with rTsASP2, and more cleavage fragments were observed after 4 h of incubation (lane 8). However, cleavage of human Hb was not observed at 4 h after incubation with rTsASP2 at the optimal pH 4.5 (S2 Fig). Both the heated anti-rTsASP2 serum and pepstatin A could inhibit the hydrolytic activity of rTsASP2 on mouse Hb (**Fig 4D**).

**Fig 5:**
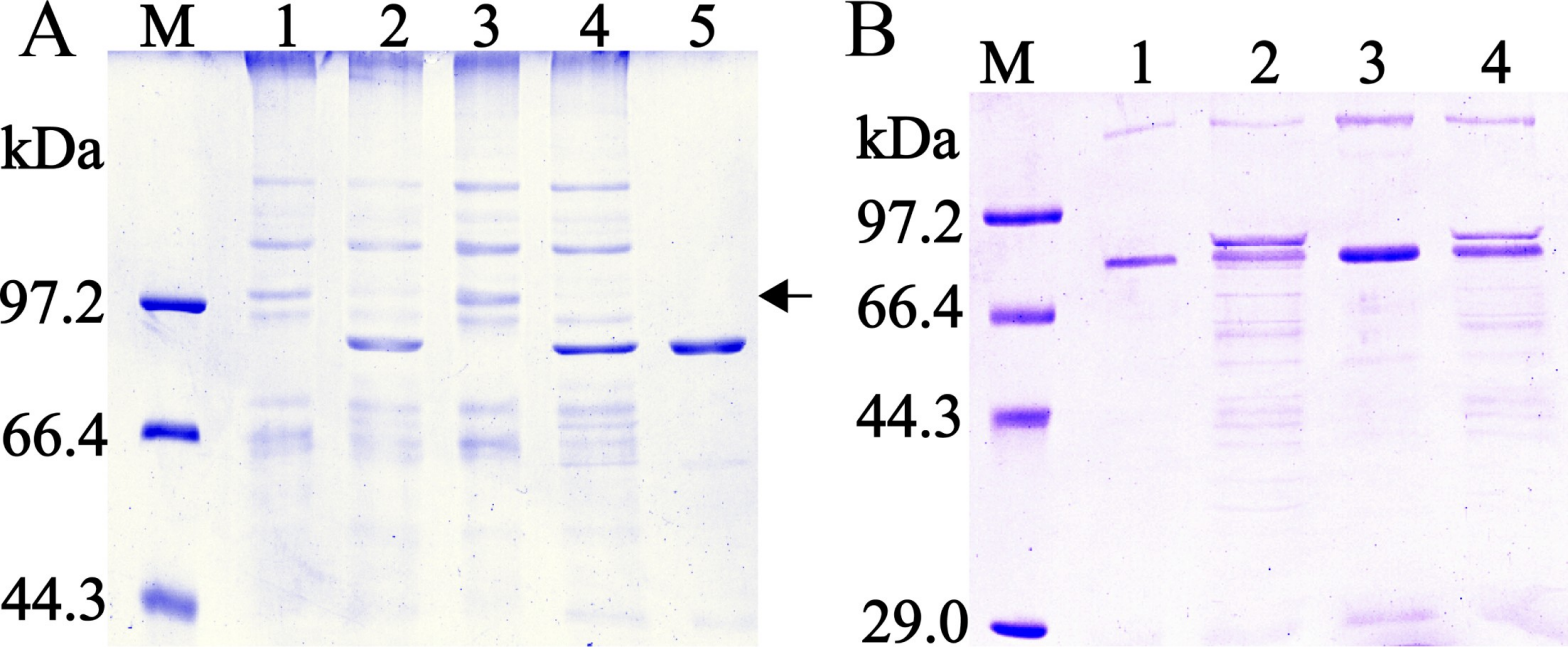
Digestion of collagen IV and IgM. The substrate including collagen IV (A) and IgM (B) was incubated with rTsASP2. The band of approximately 98-kDa collagen IV was degraded (arrow), and degraded IgM fragments were observed compared with untreated IgM. M: protein marker; lanes 1 and 3: substrates in buffer alone; lanes 2 and 4: substrate + rTsASP2; lane 5: purified rTsASP2; lanes 1 and 3: pH 2.5; lanes 2 and 4: pH 3.5.

To investigate the putative proteolytic activity of rTsASP2, several other proteins (collagen, IgM, IgG and albumin) were used as the substrate for the enzymatic catalysis assay. **Fig 5** shows that collagen (A) and IgM (B) were also hydrolyzed by rTsASP2 at pH 2.5–3.5, while no degradation of IgG and albumin was observed (S3 Fig and S4 Fig).

**Fig 6:**
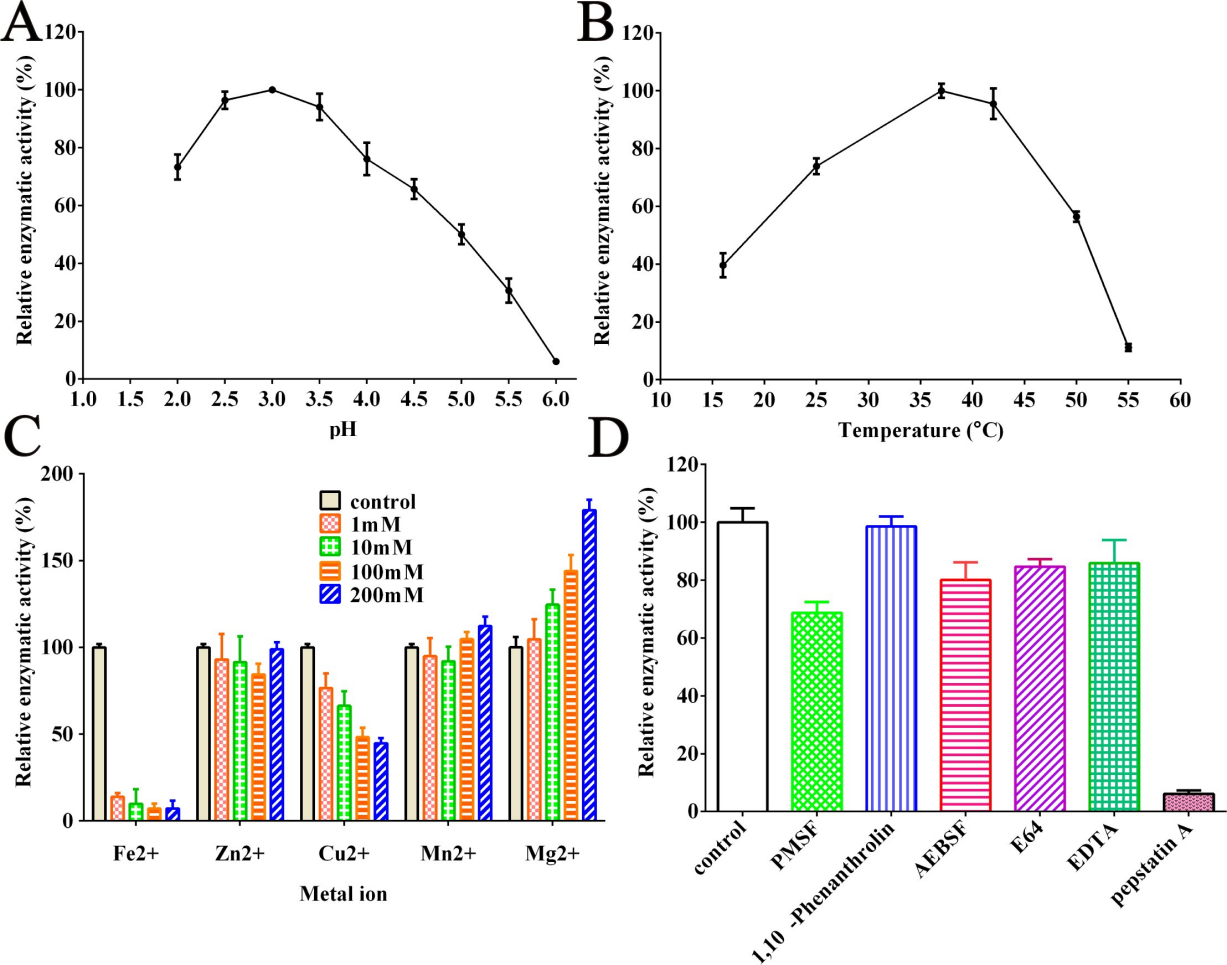
Enzymatic activity assay via cleavage of fluorescence substrate. **A:** The optimal pH of rTsASP2 enzymatic activity. B: The enzymatic activities were assayed at 15–55°C. C: The effects of metal ions on enzymatic activities. The assays were carried out under different metal ion concentrations. D: The effects of various inhibitors on enzymatic activities, where the concentration of inhibitors was 1 mM of PMSF, 1 mM of 1,10-phenanthrolin, 1 mM of AEBSF, 10 μM of E64, 1 mM of EDTA, and 10 μM of pepstatin A. All the enzymatic activities were expressed as the relative activity of the highest reaction in each experiment.

The enzymatic activity of rTsASP2 was further assessed by using synthetic fluorogenic peptide as a substrate (**Fig 6**). The maximum activity of rTsASP2 was detected at pH 3.0, although the enzyme showed a relatively broad pH range (pH 2.0–5.5) for hydrolysis of the substrate. Different metal irons have different effects on rTsASP2 catalytic activity. The enzymatic activity was clearly inhibited by the Fe^2+^ at 1 mM; it was also inhibited by Cu^2+^ in a dose-dependent manner. However, no obvious changes in hydrolytic activity were observed following the addition of Zn^2+^ or Mn^2+^ to the assay environment. Conversely, Mg^2+^ could enhance the proteolytic activity of rTsASP2, also in a dose-dependent manner. Under optimal assay conditions, rTsASP2 enzymatic activity was significantly inhibited by pepstatin A.

### TsASP2 mRNA and protein expression level after silencing the TsASP2 gene

**Fig 7:**
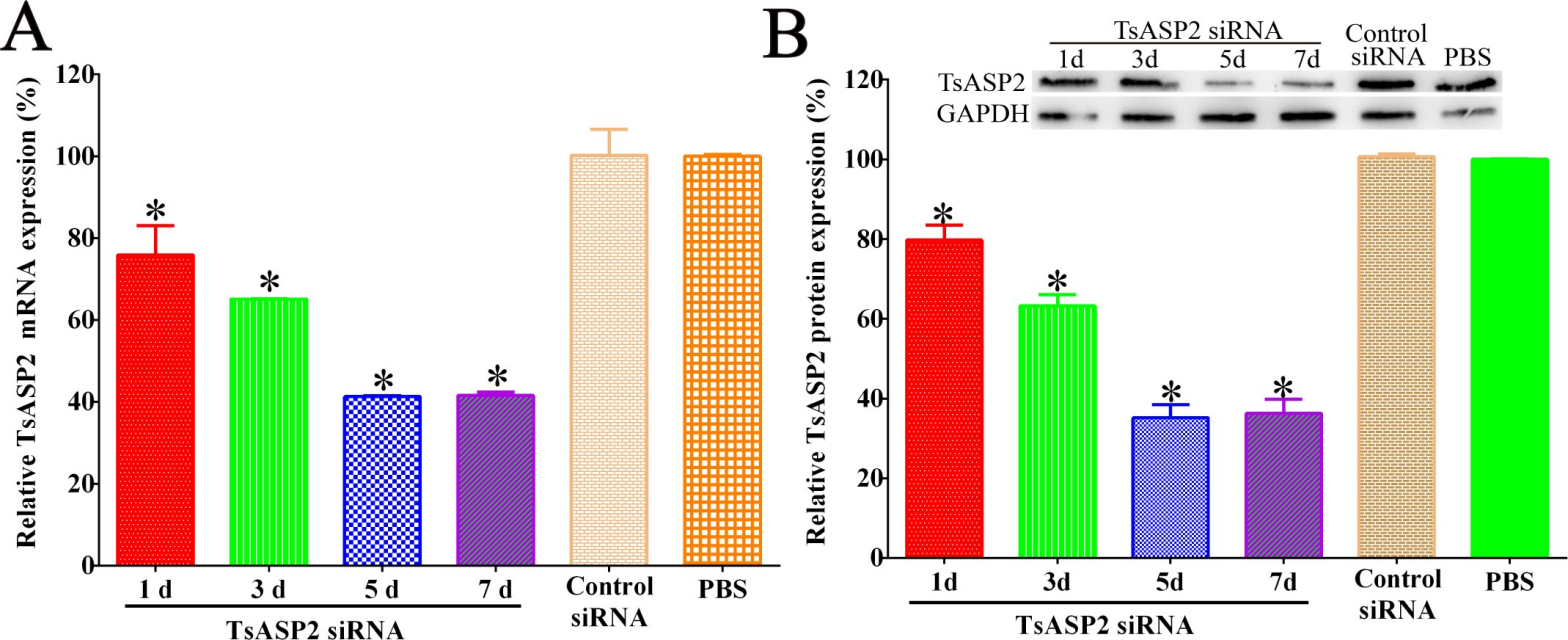
**Effects of TsASP2 RNAi on TsASP2 mRNA expression (A) and TsASP2 protein expression (B).** qPCR (A) and western blot (B) analysis of relative TsASP2 expression levels after *T*. *spiralis* muscle larvae were transfected with 5 μM TsASP2 siRNA for 1–7 d. Asterisks indicate a statistically significant difference compared with the control siRNA and PBS groups (*P < 0.05).

After 5 μM TsASP2 siRNA was delivered into worms for 5 days, the relative expression of TsASP2 mRNA and protein was reduced by 36.42% and 35.21% compared with the PBS group, respectively (**Fig 7**) (*P* < 0.05). Another siRNA of *T*. *spiralis* aspartic protease (TsASP1 siRNA) did not reduce the TsASP2 expression. Likewise, no obvious changes in TsASP1 expression were detected in worms treated with TsASP2 siRNA (S5 Fig).

### RNAi-mediated reduction of aspartic protease activity

**Fig 8:**
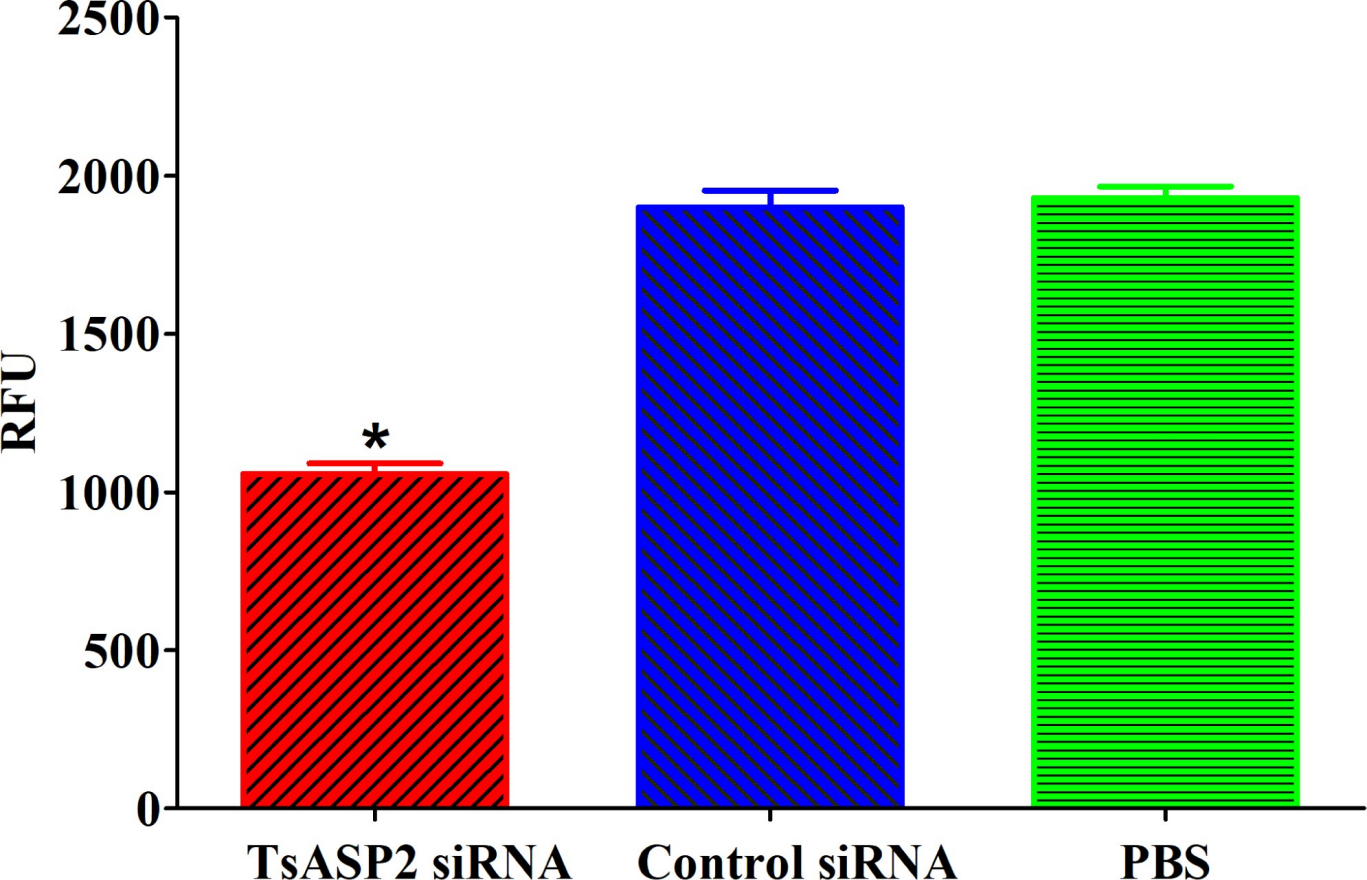
Effects of TsASP2 RNAi on aspartic protease enzymatic activities of ML. Aspartic protease activity in crude proteins of *T*. *spiralis* ML treated with siRNA was detected with a fluorogenic substrate. Asterisks indicate a statistically significant difference compared with the control siRNA and PBS groups (*P < 0.05).

After silencing the TsASP2 gene in *T*. *spiralis* ML, we investigated the activity of aspartic protease in crude protein from siRNA treated-ML using the synthetic fluorogenic peptide as the substrate. The results showed that the enzymatic activity was reduced in the TsASP2 siRNA-treated group by 54.82% compared with the PBS group. However, the enzymatic activity in the control siRNA-treated group was similar to the PBS group, suggesting that TsASP2 expression was closely related to aspartic protease in crude protein from ML (**Fig 8**).

### RNAi effect on larval penetration of IEC

**Fig 9:**
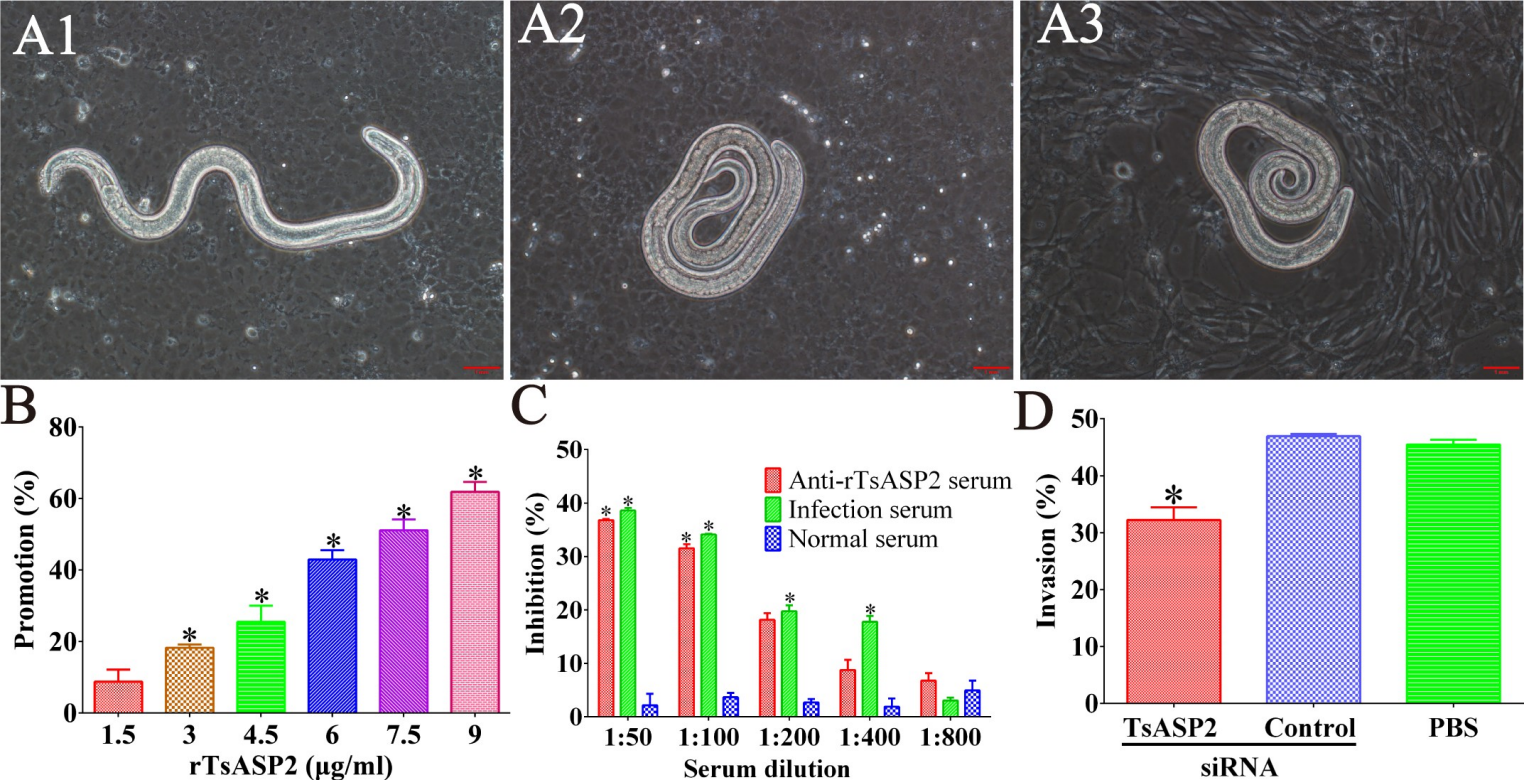
The invasion process of IECs by *T*. *spiralis* worms and RNAi inhibition on larval invasion of IEC. **A1:** The larva that invaded the IEC monolayer was mobile, and its migrating trail was observed. **A2:** The non-invaded larva was coiled. **A3:** Non-invaded larva in the C2C12 monolayer. **B:** Promotion of larval penetration of IECs by different concentrations of rTsASP2 protein, where significant differences (*P* < 0.05) are marked with asterisks (*) relative to the blank control group without rTsASP2. **C:** Inhibition of larval invasion of IECs by different dilutions of anti-rTsASP2 serum and infection serum, where significant differences (*P* < 0.05) are marked with asterisks (*) relative to the normal serum group. **D:** Larval penetration of IECs by worms treated with siRNA. Scale bar: 1 mm.

After incubation with IEC cell monolayers in semisolid medium for 2 hours, IIL invasion and migration in the monolayers were assessed (**Fig 9A**). The percentage of larval penetration was dose-dependently related to rTsASP2, exhibiting an increasing trend along with the increasing concentration of rTsASP2 protein (*F* = 353.945, *P<* 0.0001) (**Fig 9B**). When the medium was replenished with 1:100 dilutions of anti-rTsASP2 serum, infection serum or normal serum and incubated for 2 hours, the invasion rate was 35.66, 34.33 and 50.17%, respectively (χ^2^ = 29.085, *P <* 0.0001). The anti-rTsASP2 serum (1:50 to 1:100 dilutions) inhibited larval penetration into the monolayer to a greater extent than normal serum (*P* < 0.0001) (**Fig 9C**).

Additionally, silencing of TsASP2 with TsASP2 siRNA significantly suppressed larval invasion of IEC, exhibiting a 62.54% decrease when the worms were treated with 5 μM TsASP2-siRNA (χ^2^ = 13.926, *P* < 0.0001) (**Fig 9D**). No apparent reduction of larval penetration was observed when the worms were treated with control siRNA.

### TsASP2 protein is present in damaged cells invaded by larvae

**Fig 10:**
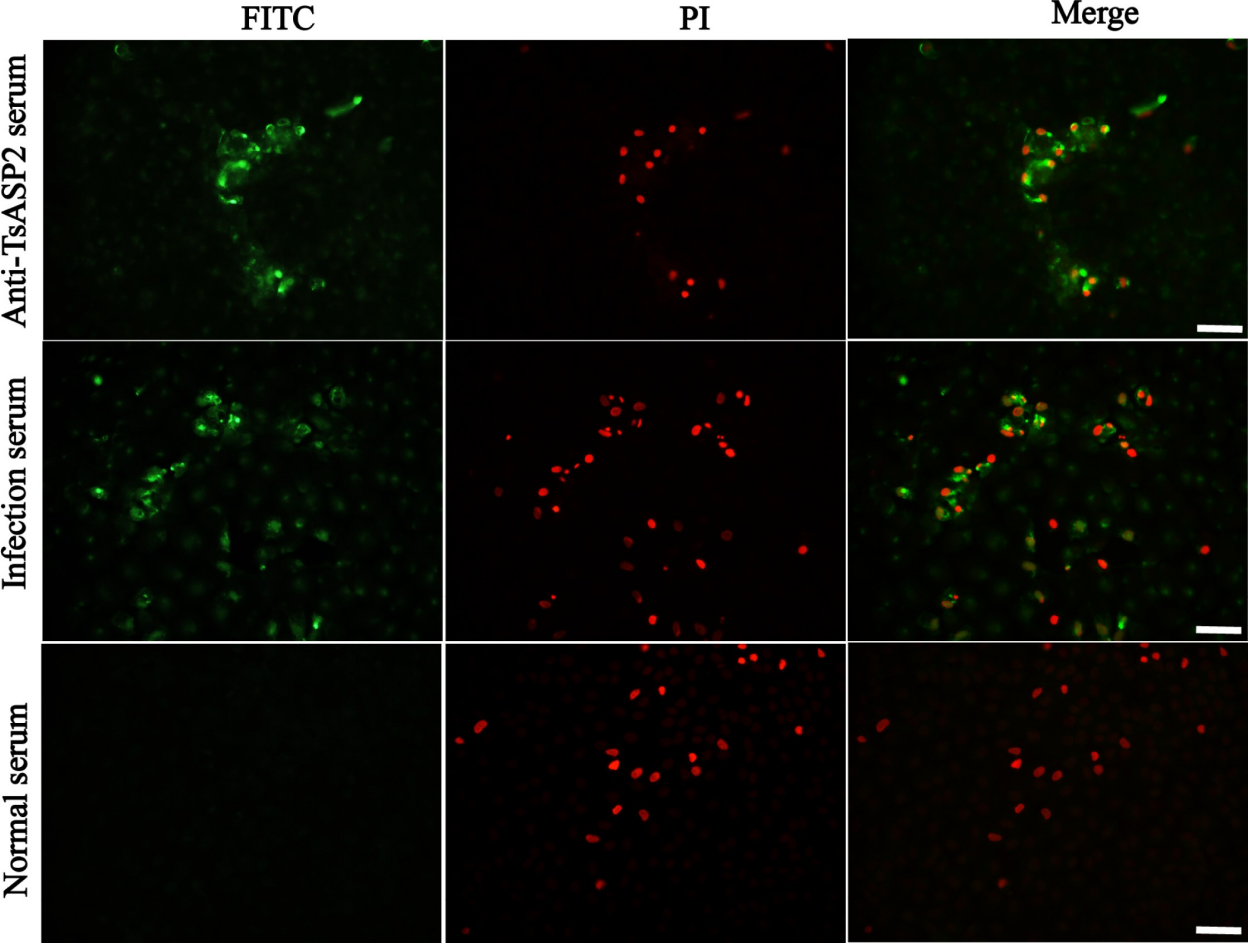
Microscopy of damaged cells following larval penetration. The IEC monolayer was stained with PI (red), and the antigen present on the damaged cells was detected with anti-rTsASP2 serum and infection serum and visualized as green fluorescence. No immunostaining was observed with normal serum. Scale bar: 50 μm.

After the invasion assay, TsASP2 protein was detected in remnants of damaged cells (stained with PI) using anti-rTsASP2 serum. In addition, secreted proteins from *T*. *spiralis* IIL larvae were also recognized in damaged cells using infection serum but not normal serum (**Fig 10**).

### RNAi effect of cell damage on the IEC monolayer

**Fig 11:**
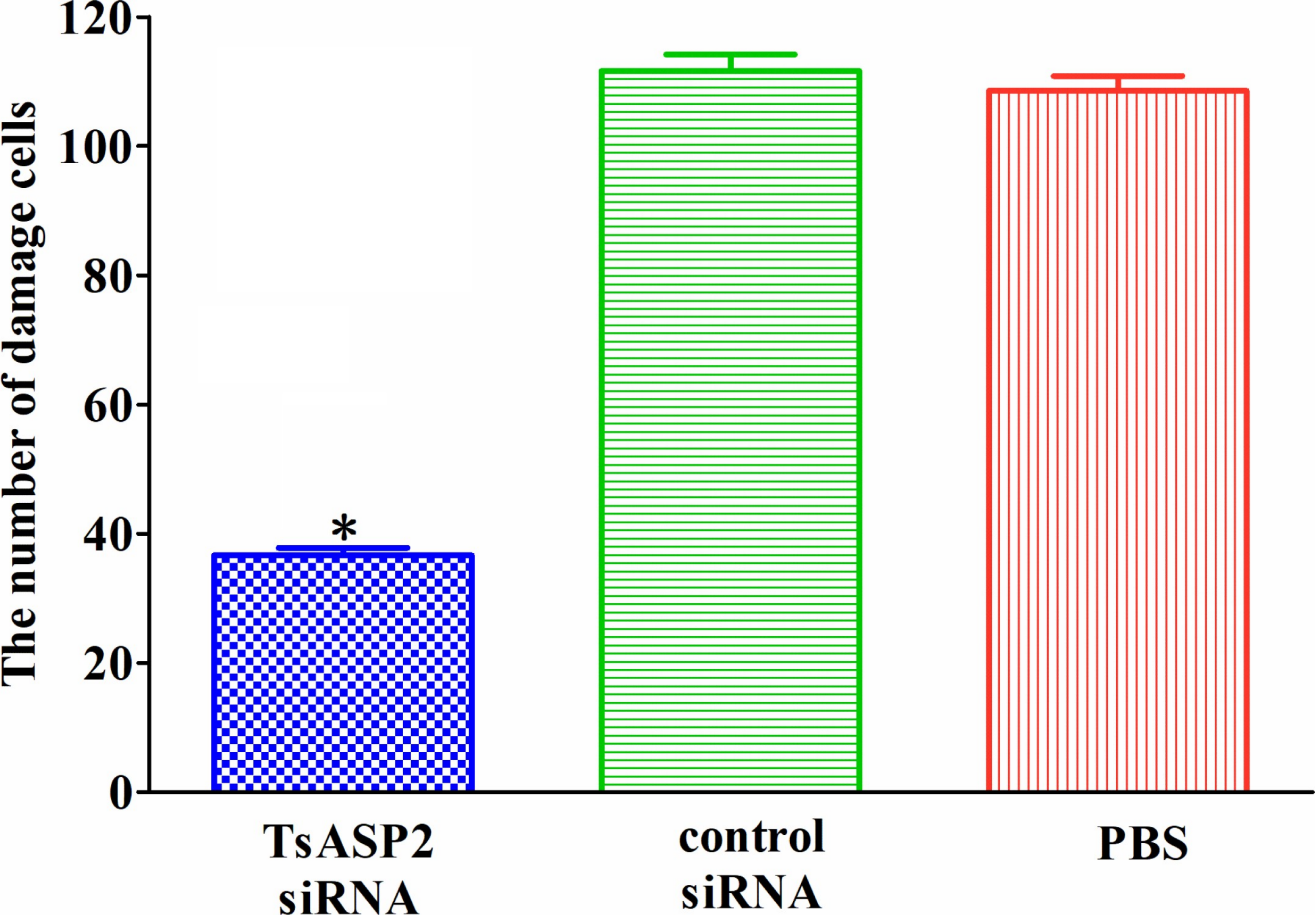
The number of damaged cells. The number of damaged cells caused by larvae treated with TsASP2 siRNA, control siRNA or PBS is expressed as the mean ± SD in an area of 3.02 mm^2^ (10×objective) for three monolayers. Asterisks indicate a statistically significant difference compared with the control siRNA and PBS groups (*P <0.05).

Larvae treated with TsASP2 siRNA, control siRNA or PBS were used in the invasion assay. Compared with the PBS or control siRNA group, the damaged cells were significant reduced in each monolayer of the TsASP2 siRNA group, implying an important role of TsASP2 in invading IECs (*P* < 0.0001) (**Fig 11).**

### RNAi effect on larval infectivity and female worm fecundity

**Fig 12:**
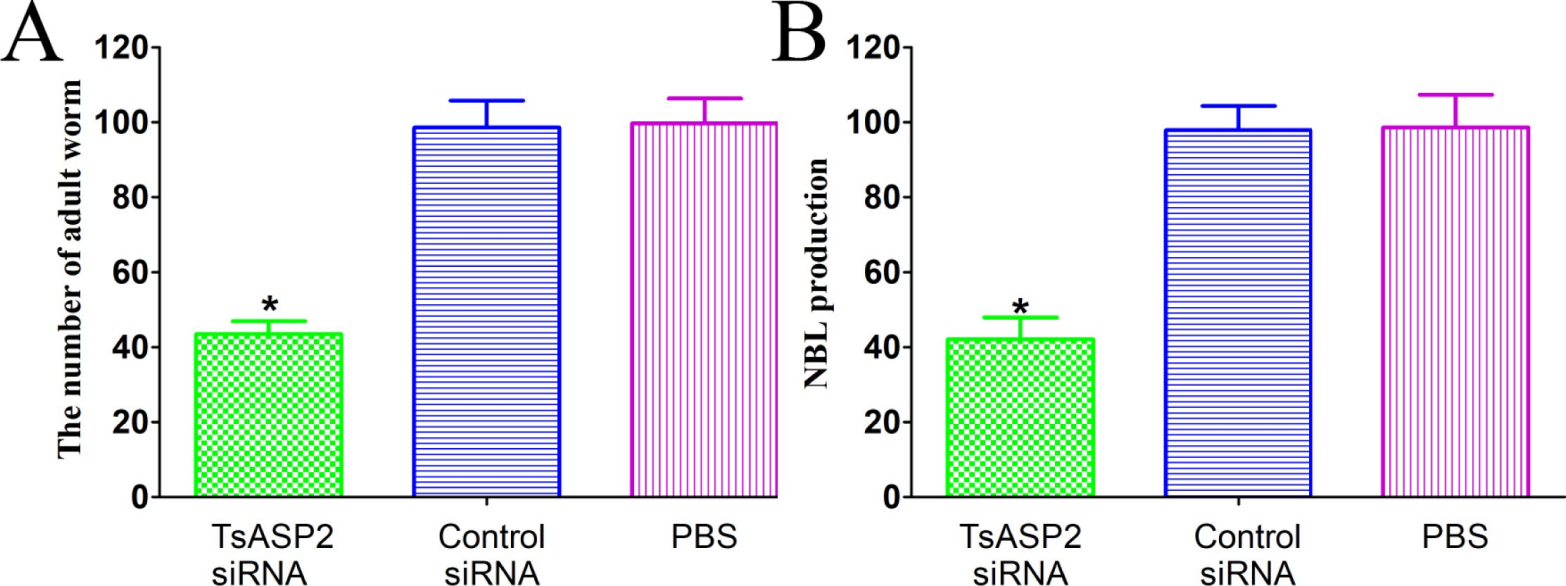
**Adult worm burdens (A) and newborn larva produced by females (B) recovered from mice infected with worms treated with TsASP2 siRNA.** Asterisks indicate a statistically significant difference compared with the control siRNA and PBS groups (*P <0.05).

**Fig 13:**
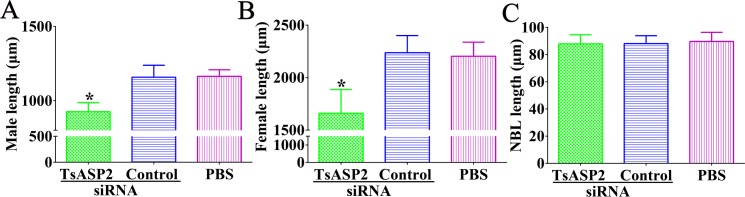
The lengths of different *T*. *spiralis* stage worms in mice infected with muscle larvae treated with TsASP2 siRNA. **A:** Male adults**; B:** female adults**; C:** NBL. Asterisks indicate a statistically significant difference compared with the control siRNA and PBS groups (*P <0.05).

Compared with the PBS group, mice that were orally infected with larvae treated with TsASP2 siRNA displayed a 56.36% reduction in adult worm burden (*F* = 260.322, *P* < 0.0001) (**Fig 12A**). The female worms collected from the TsASP2 siRNA-treated group produced fewer NBL within 72 h than those from the other two groups (*F* = 195.828, *P* < 0.0001) (**Fig 12B**). In addition, the adult worms and NBL from the three groups were morphologically observed under a microscope and their lengths measured. The length of adult worms was significantly shorter compared with the PBS group (*F*_female_ = 58.706, *F*_male_ = 41.308, *P* < 0.0001), and the length of NBL showed no significant differences among the three groups (**Fig 13**).

## Discussion

Aspartic proteases have been found in many nematodes [24,44,45] and shown to play a key role in worm invasion and survival. Park et al. [11] characterized a *T*. *spiralis* aspartic protease of 45 kDa, but its function remained unclear.

In the present study, rTsASP2 was expressed using two different plasmids (pQE-80L and PMAL-C2X) in a prokaryotic expression system. The rTsASP2 carrying a His-tagged protein was expressed in pQE-80L as an inclusion body (S1 Fig) and did not show any protease activity, so it was used only to immunize mice to obtain anti-rTsASP2 serum. In contrast, the rTsASP2 co-expressed with the MBP tag was mainly used to investigate its enzymatic activity and biological function.

The expression of TsASP2 in various developmental stages of *T*. *spiralis* was obviously different. Native TsASP2 protein in crude proteins of all the worm stages except NBL was detected by anti-rTsASP2 serum. Furthermore, no immuno-staining was observed in NBL by IFA, which confirmed the low level of TsASP2 expression in this stage, suggesting that TsASP2 protein expression in the NBL stage was too low to be detected by Western blotting and IFA. The IFA results revealed that TsASP2 was located in the hindgut, midgut and muscle cells of ML and IIL, suggesting that TsASP2 might participate in nutrient intake. The aspartic proteases of other parasites are also located in the intestine and have been suggested to have essential functions in parasite nutrition [21,23]. The strong staining encircling the embryos suggested that TsASP2 might also be involved in female reproduction.

The primary function of aspartic protease is to digest hemoglobin [46]. In our study, rTsASP2 hydrolyzed human and murine Hb. Similar to other aspartic proteases, rTsASP2 also cleaves Hbs at an acidic pH (pH 2.5–4.5), with diverse pH values for different Hbs. The different optimal pH of aspartic protease activity could be related to different substrates [47,48]. The host-specific cleavage of Hbs by aspartic protease has been verified in previous studies [20,21]. Similarly, rTsASP2 cleaved mouse Hbs more efficiently than human Hbs. The high degradation efficiency of murine Hbs was likely due to the passaging of the *T*. *spiralis* in mice in our laboratory for more than 30 years. In addition, rTsASP2 could also hydrolyze IgM and collagens at an acidic pH, which may be associated with immune evasion, degradation of host proteins and larval migration through host tissues [49,50]. Given the highest TsASP2 expression level in the IIL stage, TsASP2 might play a key role in *T*. *spiralis* invasion of intestinal epithelium.

The enzymatic activity of rTsASP2 was further characterized by hydrolyzing the fluorescent substrate at various condition (as shown in Fig 6). The optimal pH for rTsASP2 was 3.0, which suggested that rTsASP2 had hydrolytic activity under acidic conditions similar to its optimal pH for cleaving Hb. The enzymatic activity of rTsASP2 was inhibited by Fe^2+^ and Cu^2+^ but enhanced by Mg^2+^, and it was not sensitive to Zn^2+^ and Mn^2+^. Previous studies have demonstrated that the activity of aspartic protease (ASP) from parasites is sensitive to copper, such as ASP from *Trichomonas vaginalis* (TV-CatD) and *Plasmodium falciparum* (plasmepsin II AP) [51,52]. In another study on *Metschnikowia pulcherrima* ASP [53], the cations Fe^2+^ and Mg^2+^ were found to be insensitive to ASP, while Mn^2+^ and Zn^2+^ had a slight inhibitory effect on enzymatic activity. The distinct properties of TsASP2 from other ASPs were likely due to their origination from different organism species. Nevertheless, elucidation of the effects of metal ions on enzymatic activity of ASP requires further investigation.

The enzymatic activity of rTsASP2 was clearly inhibited by pepstatin A (a common ASP inhibitor), suggesting that TsASP2 is a kind of typical aspartic protease [52]. Additionally, PMSF could also suppress 68.5% of the enzymatic activity, which differed from ASP purified form *Plasmodium vivax*. No obvious inhibition was detected using other enzyme inhibitors, similar to other aspartic proteases [54,55]. The proteolytic activities of TsASP2 were further confirmed by the RNAi assay. At five days after the ML were treated with 5 μM TsASP2 siRNA, the TsASP2 mRNA and protein expression levels were significantly reduced, suggesting that the TsASP2 gene was successfully silenced. Furthermore, aspartic protease activity was significantly reduced in siRNA-treated worm crude proteins, demonstrating that TsASP2 protein expression was associated with the proteolytic activities of *T*. *spiralis* aspartic protease.

In previous studies, protease enzymatic activities have been suggested to play key roles in *T*. *spiralis* infection, especially larval invasion of host IECs. The results of the larval *in vitro* invasion assay demonstrated that TsASP2 promoted larval penetration of IECs, and the percent of invaded larvae was dose-dependent on rTsASP2. It has been reported that anti-serum against Na-APR-2 (a hookworm aspartic protease) can inhibit the migration of the parasite through skin [20]. Similarly, larval invasion of IECs was significant inhibited when anti-rTsASP2 serum was added to the medium, supporting a facilitating function of TsASP2 in *T*. *spiralis* invasion into IECs. Our previous studies have shown that recombinant serine proteases also have a promoting role in larval invasion of IECs, whereas antibodies against serine protease have an inhibitory effect, suggesting that various proteases participate in *T*. *spiralis* larval invasion and development [15, 56–59].

RNAi is commonly applied to downregulate target molecules and investigate the biological functions of target proteins [27,34]. After downregulating TsASP2 in the ML stage, and then activating the knockdown ML into IIL, the transfected larvae showed a 62.54% reduction of larval invasion. After larval invasion, the damaged cells could be clearly detected by PI staining and then counted by microscopy. The damaged cells were significantly reduced when IECs were co-incubated with larvae treated with TsASP2 siRNA. Previous studies have suggested that the evaluation of cell damage after invasion is an objective indicator for assessing the invasion efficiency [43,60]. Our results revealed that silencing TsASP2 expression could impede *T*. *spiralis* invasion into IECs *in vitro*, validating that TsASP2 participated in larval penetration of host intestinal epithelium.

The TsASP2 function in *T*. *spiralis* penetration of IECs was further confirmed by silencing the TsASP2 gene with RNAi. Previous results have demonstrated that silencing of some *T*. *spiralis* genes with RNAi can impair *T*. *spiralis* worm viability [7] or inhibit its development and reproductive capacity [33, 34,61]. Silencing of the expression of these genes in *T*. *spiralis* results in reduced parasite viability and infectivity, such as impaired *T*. *spiralis* molting or invasion. Our results revealed low enteral adult worm burdens, and NBL production of female worms was reduced when TsASP2 was silenced, indicating that enteral larval growth, development and female fecundity were suppressed. Silencing of the TsASP2 gene also impeded other *T*. *spiralis* developmental stages, as adult worms recovered from mice infected with siRNA-treated larvae were shorter than those recovered from the control and PBS groups. Specific gene silencing by RNAi has been widely applied for gene function identification in other parasites [62,63], and the results of the present study indicated that TsASP2 played a crucial role in *T*. *spiralis* invasion of IEC, and silencing of the TsASP2 gene significantly reduced larval infectivity and development in mice.

In conclusion, TsASP2 was highly expressed in the IIL stage of *T*. *spiralis*, mainly located in hindgut, midgut and muscle cells of ML and IIL and around intrauterine embryos of female adults. The TsASP2 has the native aspartic protease activities to cleave Hbs, IgM and collagen under acidic condition, and the proteolytic activity was host-specific. Silencing of TsASP2 gene by RNAi could significantly reduce the TsASP2 protein expression, which inhibited the native aspartic protease activities and larval invasion of host’ enterocytes. The results indicated that TsASP2 plays an important role in the *T*. *spiralis* invasion and it could be a candidate vaccine target molecular against *T*. *spiralis* infection.

## Supporting information

S1 Fig SDS-PAGE analysis of rTsASP2. (DOCX)

S2 Fig Hydrolysis efficiency effect of rTsASP2 on human Hb (pH 4.5). (DOCX)

S3 Fig rTsASP2 has no degradation on IgG from human (A) and mice (B).
(DOCX)

S4 Fig rTsASP2 has no degradation on albumins from human (A) and bovine (B).
(DOCX)

S5 Fig qPCR (A) and Western blot (B) analysis of the expression levels of TsASP1 and TsASP2 in ML transfected using TsASP1 siRNA or TsASP2 siRNA.
(DOCX)
