# Supplementary material for: Molecular characterization of a Trichinella spiralis aspartic protease and its facilitation role in larval invasion of host intestinal epithelial cells
Source: PLoS Negl Trop Dis. 2020 Apr 27;14(4):e0008269. doi: 10.1371/journal.pntd.0008269 (PMC7205320; doi:10.1371/journal.pntd.0008269)
Supplement: S1 Fig — (DOCX) [file pntd.0008269.s001.docx]

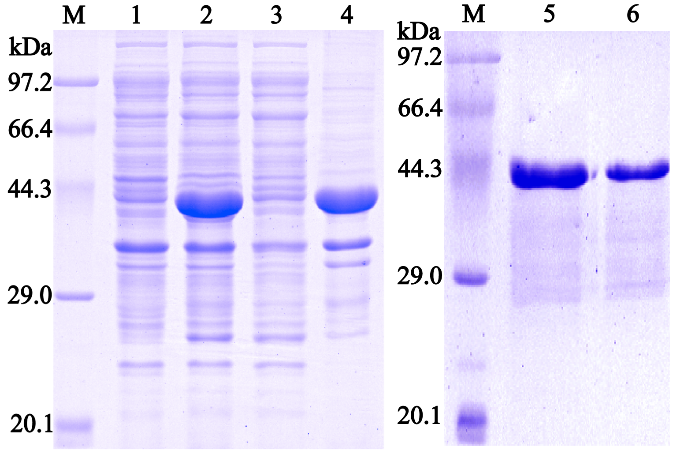


**S1 Fig. SDS-PAGE analysis of rTsASP2.** M: protein marker; lane 1: lysates of recombinant bacteria incorporating pQE-80L/TsASP2 without induction; lane 2: lysates of recombinant bacteria incorporating pQE-80L/TsASP2 after induction; lane 3: lysate supernatant of recombinant bacteria incorporating pQE-80L/TsASP2 after induction; lane 4: sediment of recombinant bacteria incorporating pQE-80L/TsASP2 after induction; lane 5, 6: purified rTsASP2.
