# Supplementary material for: Molecular characterization of a Trichinella spiralis aspartic protease and its facilitation role in larval invasion of host intestinal epithelial cells
Source: PLoS Negl Trop Dis. 2020 Apr 27;14(4):e0008269. doi: 10.1371/journal.pntd.0008269 (PMC7205320; doi:10.1371/journal.pntd.0008269)
Supplement: S2 Fig — (DOCX) [file pntd.0008269.s002.docx]

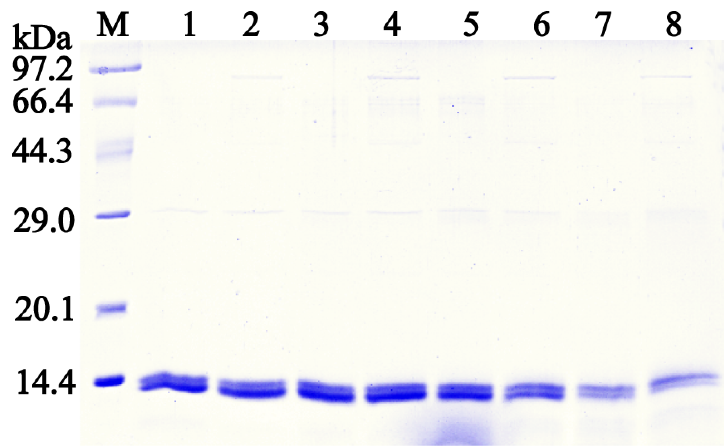


**S2 Fig. Hydrolytic efficiency effect of rTsASP2 on human Hb (pH 4.5).** M: protein marker; lanes 1, 3, 5 and 7: Hb alone; lanes 2, 4, 6 and 8: Hb+ rTsASP2; lanes 1 and 2: 5 min; lanes 3 and 4: 30 min; lanes 5 and 6: 90 min; lanes 7 and 8: 4 h**.**
