# Supplementary material for: Molecular characterization of a Trichinella spiralis aspartic protease and its facilitation role in larval invasion of host intestinal epithelial cells
Source: PLoS Negl Trop Dis. 2020 Apr 27;14(4):e0008269. doi: 10.1371/journal.pntd.0008269 (PMC7205320; doi:10.1371/journal.pntd.0008269)
Supplement: S4 Fig — rTsASP2 has no degradation on albumins from human (A) and bovine (B). (DOCX) [file pntd.0008269.s004.docx]

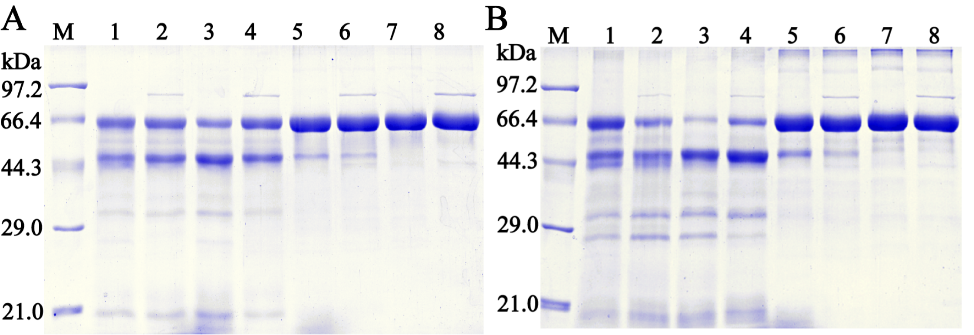


**S4 Fig. rTsASP2 has no degradation on albumins from human (A) and bovine (B).** M: protein marker; lanes 1, 3, 5 and 7: albumin alone; lanes 2, 4, 6 and 8: albumin + rTsASP2; lanes 1 and 2: pH 2.5; lanes 3 and 4: pH 3.5; lanes 5 and 6: pH 4.5; lanes 7 and 8: pH 5.5.
