# Supplementary material for: Molecular characterization of a Trichinella spiralis aspartic protease and its facilitation role in larval invasion of host intestinal epithelial cells
Source: PLoS Negl Trop Dis. 2020 Apr 27;14(4):e0008269. doi: 10.1371/journal.pntd.0008269 (PMC7205320; doi:10.1371/journal.pntd.0008269)
Supplement: S5 Fig — qPCR (A) and Western blot (B) analysis of the expression levels of TsASP1 and TsASP2 in ML transfected using TsASP1 siRNA or TsASP2 siRNA. (DOCX) [file pntd.0008269.s005.docx]

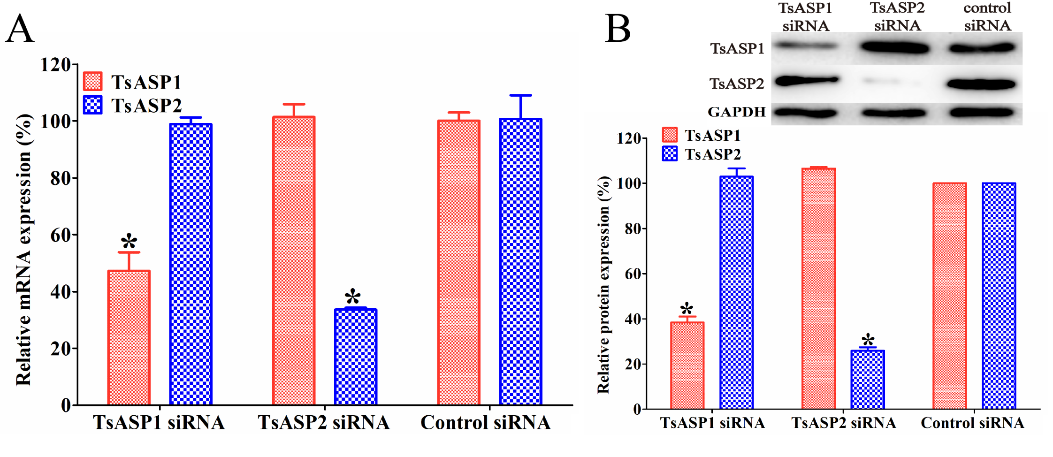


**S5 Fig. qPCR (A) and Western blot (B) analysis of expression levels of TsASP1 and TsASP2 in ML transfected using TsASP1 siRNA or TsASP2 siRNA.** Asterisks indicate a statistically significant difference compared with the control siRNA (**P* < 0.05).
